# Common Genetic Variation in *CYP17A1* and Response to Abiraterone Acetate in Patients with Metastatic Castration-Resistant Prostate Cancer

**DOI:** 10.3390/ijms17071097

**Published:** 2016-07-09

**Authors:** Moritz Binder, Ben Y. Zhang, David W. Hillman, Rhea Kohli, Tanvi Kohli, Adam Lee, Manish Kohli

**Affiliations:** 1 Department of Internal Medicine, Mayo Clinic, 200 First Street SW, Rochester, MN 55905, USA; Binder.Moritz@mayo.edu; 2 Department of Oncology, Mayo Clinic, 200 First Street SW, Rochester, MN 55905, USA; Zhang.Ben@mayo.edu; 3 Division of Biomedical Statistics and Informatics, Mayo Clinic, 200 First Street SW, Rochester, MN 55905, USA; Hillman.David@mayo.edu; 4 Mayo High School, 1420 11th Avenue SE, Rochester, MN 55904, USA; rheak99@gmail.com (R.K.); tankoh98@gmail.com (T.K.); 5 Experimental and Clinical Pharmacology Department, University of Minnesota, 515 Delaware St. SE, Minneapolis, MN 55455, USA; leeam@umn.edu

**Keywords:** metastatic castration-resistant prostate cancer, abiraterone acetate, single nucleotide polymorphism, *CYP17A1*, predictive biomarker

## Abstract

Treatment with abiraterone acetate and prednisone (AA/P) prolongs survival in metastatic castration-resistant prostate cancer (mCRPC) patients. We evaluated the genetic variation in *CYP17A1* as predictive of response to AA/P. A prospective collection of germline DNA prior to AA/P initiation and follow-up of a mCRPC cohort was performed. Five common single-nucleotide polymorphisms (SNPs) in *CYP17A1* identified using a haplotype-based tagging algorithm were genotyped. Clinical outcomes included biochemical response and time to biochemical progression on AA/P. Logistic regression was used to assess the association between tag SNPs and biochemical response. Proportional hazards regression was used to assess the association between tag SNPs and time to biochemical progression. Odds or hazard ratio per minor allele were estimated and *p*-values below 0.05 were considered statistically significant. Germline DNA was successfully genotyped for four tag SNPs in 87 patients. The median age was 73 years (54–90); the median prostate-specific antigen was 66 ng/dL (0.1–99.9). A single SNP, rs2486758, was associated with lower odds of experiencing a biochemical response (Odds ratio 0.22, 95% confidence interval 0.07–0.63, *p* = 0.005) and a shorter time to biochemical progression (Hazard ratio 2.23, 95% confidence interval 1.39–3.56, *p* < 0.001). This tag SNP located in the promoter region of *CYP17A1* will need further validation as a predictive biomarker for AA/P therapy.

## 1. Introduction

Prostate cancer is the second leading cause of cancer-related mortality in patients in the United States, with an estimated 26,120 deaths in 2016 [1]. Almost all prostate cancer–related deaths occur in patients with advanced, metastatic disease. The initial treatment for this stage of the disease is androgen deprivation therapy, which provides effective control of the disease for variable time periods in patients with advanced metastatic hormone-sensitive prostate cancer by depleting testosterone [2,3,4]. Inevitably, disease progression occurs to a castration-resistant state (mCRPC) [5,6,7,8], although there is considerable variability in time to progression reflecting an underlying genetic heterogeneity. Once a patient has progressed to mCRPC, the median overall survival (OS) is limited to 15–35 months, despite the availability of several therapeutic options [9,10,11,12,13]. Recent therapeutic advances including the successful targeting of the testosterone-androgen receptor (AR) axis with several new drugs have resulted in slowing castration-resistant disease progression and improved survival. One of the novel drugs contributing to the increase in survival in the castration-resistant stage is the cytochrome P45017 (CYP17) inhibitor abiraterone acetate (AA) which has efficacy in combination with prednisone (P) in both the pre- and post-chemotherapy setting, although the response to treatment remains variable [14,15,16,17]. CYP17A1 is encoded by a single gene on chromosome 10q24.3 (*CYP17A1*), expressed in the adrenals and gonads, and a membrane-bound dual-function monooxygenase with a critical role in the synthesis of many human steroid hormones [18,19]. Defects in *CYP17A1* were found to cause 17α-hydroxylase deficiency and 17,20-lyase deficiency [18]. *CYP17A1* has also been implicated in the development of cardiovascular diseases through effects on blood pressure [20,21,22]. *CYP17A1* is an important target for the treatment of prostate cancer that proliferates in response to androgens [19,23] Genetic variation of *CPY17A1* was found to be associated with increased risk of prostate cancer [24,25,26,27,28]. Furthermore, alterations of CYP17A1 activity have been described as possible mechanisms of resistance to hormonal treatments in experimental models of prostate cancer [29].

We investigated the genetic variation in a key sex steroid pathway gene, *CYP17A1*, which results in the enzyme activity for metabolizing intracellular testosterone levels as a potential predictive factor for response to AA/P at the castration-resistant stage.

## 2. Results

The characteristics of the 87 patients identified to meet the inclusion criteria are shown in Table 1. The median time from the initiation of androgen deprivation therapy to the development of mCRPC was two years (0–17). The time from DNA collection to the initiation of AA/P was two years (0–6). Four of the five (80%) tag single nucleotide polymorphisms (SNPs) in *CYP17A1* were successfully genotyped in all patients with an adequate call rate of 0.958. No markers were out of Hardy-Weinberg equilibrium (*p* > 0.001 for all comparisons), and the mean imputation *R*^2^ was 0.974. The four successfully genotyped SNPs (rs4919685, rs743572, rs17115100, and rs2486758) provided 100% coverage of the common genetic variations (minor allele frequency ≥ 0.05) in *CYP17A1* (chromosome 10, base pairs 102,830,531–102,837,533).

The associations between the four genotyped SNPs and the biochemical response to as well as the time to biochemical progression on AA/P are shown in Table 2. The minor allele frequency for rs2486758 was 0.33 in this cohort of patients with mCRPC.

**Figure 1:**
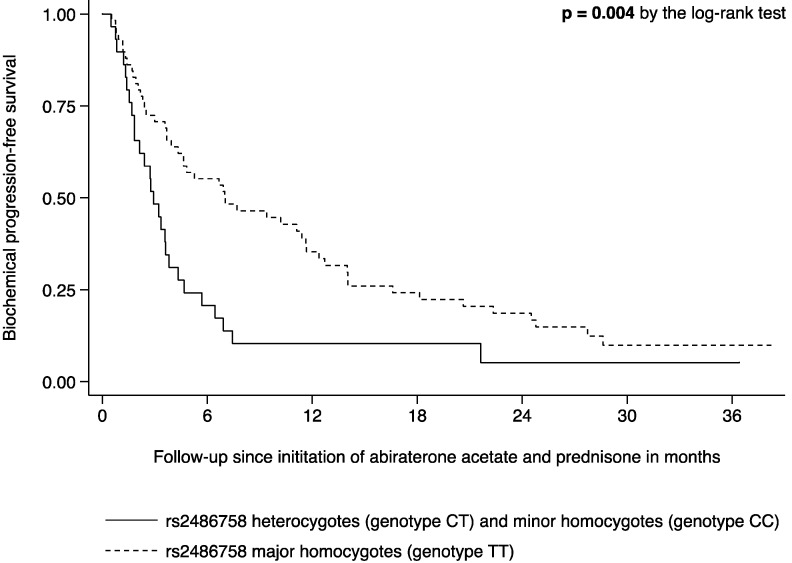
Kaplan-Meier estimates of time to biochemical progression on abiraterone acetate and prednisone stratified by rs2486758 genotype (hetero- and minor homozygotes versus major homozygotes).

The genetic variant rs2486758 (T>C) in *CYP17A1* was negatively associated with biochemical response to AA/P (Odds ratio 0.22, 95% confidence interval 0.07–0.63, *p* = 0.005), translating into a more-than-four-fold decrease in the odds of biochemical response to AA/P per minor allele. The biochemical response rate to AA/P was 50% in patients with wild-type rs2486758 and 17% in patients with at least one minor allele (*p* = 0.005). Unadjusted and multivariable-adjusted estimates for the effect of rs2486758 on biochemical response to AA/P are shown in Table 3. The genetic variant rs2486758 (T>C) in *CYP17A1* was also associated with a shorter time to biochemical progression on AA/P (Hazard ratio 2.23, 95% confidence interval 1.39–3.56, *p* < 0.001), translating into a more-than-two-fold increase in the hazard of biochemical disease progression on AA/P per minor allele. After a median follow-up time of 4.6 months (3.4–6.9), 77 patients (89%) had progressed. The median time to biochemical progression was 7.0 (4.3–11.6) months in patients with wild-type rs2486758 and 2.9 months (1.8–3.8) in patients with at least one minor allele (*p* = 0.004, Figure 1). Unadjusted and multivariable-adjusted estimates for the effect of rs2486758 on time to biochemical progression on AA/P are shown in Table 3.

## 3. Discussion

AA/P was studied in patients with metastatic castration-resistant prostate cancer both prior to and after administration of cytotoxic chemotherapy [14,15]. The biochemical response rates in these studies were lower in patients who received AA/P after cytotoxic chemotherapy (38%) compared to patients treated with pre-chemotherapy AA/P (62%). The observed biochemical response rate in our cohort (39%) is very similar to the expected biochemical response rate (46%), taking into account the large proportion of our patients who had been treated with prior cytotoxic chemotherapy. No predictive factors have been identified previously for response to AA/P, including the Gleason score [30] or type and duration of prior endocrine therapies [31]. Another smaller study showed a poorer response to AA/P in patients with shorter responses to previous prostate cancer therapies [32]. In our study cohort, 29 of the 87 patients (33%) receiving AA/P for mCRPC harbored the rs2486758 variant of *CYP17A1* which was associated with a risk for lower biochemical response and shorter time to biochemical progression when taking AA/P. The rs2486758 minor allele which is located in the intergene region near the 5’ of the *CYP17* gene remained statistically significant for the association after adjusting for multiple testing as well as potential confounding clinical variables including the Gleason score at the time of initial diagnosis, prostate-specific antigen at the time of initiating treatment, and whether the treatment was administered pre- or post-chemotherapy. Our data suggests a more-than-four-fold decrease in the odds of biochemical response to AA/P in patients with mCRPC per rs2486758 minor allele (C versus T). Furthermore, each minor allele was associated with a more-than-two-fold increase in the hazard ratio of biochemical disease progression. In previous studies, the rs2486758 polymorphism of *CPY17A1* was associated with a significant increased risk of prostate cancer [24,25,26,27]. A meta-analysis of these studies confirmed this association (C versus T, odds ratio 1.07, 95% confidence interval 1.03–1.12, *p* = 0.002) [28]. Mechanistically, this 7% increase in the risk of prostate cancer was attributed to the location of rs2486758 in the promoter region of *CYP17A1*. Alterations of CYP17A1 activity including up-regulation have been described as a mechanism of resistance to AA/P in human prostate cancer xenografts, making the enzyme a potential candidate as a predictive biomarker [29]. With the up-regulation of CYP17A1 being described as a mechanism of resistance to AA/P and functional studies confirming significantly increased transcriptional activity of *CYP17A1* with the minor allele (C versus T), up-regulation is a likely explanation for the observed effects [33]. Likewise, the shorter duration of the response may be explained by the up-regulation of CYP17A1 overcoming the effects of AA/P treatment. The rs2486758 minor allele has been reported to be associated with higher serum 17β-estradiol levels in premenopausal women [34], but it is unknown if sex steroids in men who have undergone castration are also altered in the presence of the polymorphism and if that will affect the response to treatment with AA/P.

Other genome-based predictive factors for AA/P that have been investigated include copy number variations of *CYP17A1* in the serum of patients with mCRPC [35]. These may have contributed to the observed effects but were not evaluated in our study, which investigated germline factors for determining response to treatment. Furthermore, in a small study, germline variation in other signaling pathways such as the *SULT1E1* variants described by Agarwal et al. [36] has been shown to have effects on duration of response and warrant validation. The aforementioned factors will need to be evaluated in concert with our findings to gain a better understanding of the different factors affecting response to treatment. Mostaghel et al. [29] also demonstrated the induction of ligand-independent androgen receptor (AR) variants in response to AA/P treatment, which is another plausible explanation for disease progression in this setting. Furthermore, differential expression of AR-V7 is another plausible explanation for the difference in observed response rates [37]. The mechanism described by Antonarakis et al. is biologically plausible, although the results have not been validated [38]. The technology to detect AR-V7 expression detection in circulating tumor cells is becoming available for clinical use, but is yet to be adopted in clinical practice.

A strength of our study was that it included prospectively followed patients after enrollment in a study devised to identify biomarkers in advanced prostate cancer in a uniform manner and therefore did not involve samples of convenience. The limitations include small sample size, lack of a validation cohort, and a candidate approach which restricts information of other germline or somatic genetic aberrations such as *AR* copy number variations and *AR* mutations on response to treatment. These will need to be evaluated in larger cohorts treated with AA/P in order to develop predictive models for individualizing treatment with AA/P in advanced prostate cancer.

## 4. Materials and Methods

### 4.1. Cohort and Clinical Data

This study included patients with mCRPC who were enrolled in a prospective and uniform collection of bio-specimens at Mayo Clinic (Rochester, MN, USA); the study was approved by the institutional review board (IRB# 09-007355). Written consent was obtained from all patients prior to enrollment. The prostate cancer cohort in this hospital-based registry includes patients with advanced-stage prostate cancer who provide blood and urine specimens at the time of enrollment for the purpose of biomarker identification to treatments and are followed for outcomes including overall survival as has been previously reported in detail [39,40,41]. All patients were enrolled between September 2009 and December 2012 and followed for outcomes after enrollment. The study involves collection of blood and urine specimens including the collection of peripheral blood mononuclear cells, which were used to extract germline DNA. Only those patients receiving the combination of AA/P for mCRPC stage were included in this analysis. Patients with missing clinical or unavailable genetic information were excluded from the analysis if they had received this drug combination. All patients had follow-up data abstracted from medical records for treatment and disease outcomes. Patient characteristics collected included demographic information, age at the time of DNA specimen collection, initial cancer diagnosis date, Gleason score at the time of initial diagnosis, time between initial prostate treatment and initiation of castration for mCRPC stage after failure of androgen deprivation therapy, date of castration, date of progression while receiving AA/P for mCRPC, prostate-specific antigen at the time of disease progression while receiving AA/P. In addition, we also collected relevant clinical information before androgen deprivation therapy, including stage at the time of initial prostate cancer diagnosis and primary prostate treatments received previously. Follow-up and care for all patients was performed by physicians specializing in prostate cancer as per the standard of care.

### 4.2. Genetic Data

Germline DNA was purified from buffy coats isolated from whole blood obtained from the study patients. Briefly, germline DNA was extracted from buffy coats isolated from whole blood obtained from the identified (or selected) patients using a standardized organic (phenol-chloroform) purification procedure. Incubation of RNase A was also used to remove any contaminating RNA. Following extraction, DNA quality and quantity was assessed using spectrophotometry and the picogreen assay. All DNA samples utilized for genotyping required 260/280 spectrophotometric ratios of ≥1.8. Double stranded DNA concentrations obtained from the picogreen assay were all ≥50 ng/µL. DNA samples were diluted to a working concentration of 20 ng/µL prior to genotyping using the MassARRAY platform. Forty-eight SNPs in five candidate genes *CYP17A1*, *HSD3B2*, *ESR1*, *ESR2*, and *AR* were identified using a haplotype-based tagging algorithm [42]. Genotyping of these tag SNPs using the MassARRAY platform (Agena Bioscience, San Diego, CA, USA) was performed at the Mayo Clinic Genotyping Facility. The MaCH imputation program [43] was used to carry out multiple genotype imputation for missing genotypes using the HapMap Phase II release #22 CEU reference panel [44]. The a priori hypothesis for this study was an association between common genetic variation in *CYP17A1* and biochemical response to AA/P. The four remaining candidate genes were genotyped for different outcomes in a larger cohort of men with advanced prostate cancer.

### 4.3. Outcomes

The clinical outcomes of interest included biochemical response to treatment as defined by ≥50% prostate-specific antigen decrease after initiating AA/P treatment. The second response outcome of interest for association with genotypes was time to biochemical progression as defined by >25% increase in prostate-specific antigen on two consecutive tests at least two weeks apart. Patients who had not progressed at the time of data analysis were censored.

### 4.4. Statistical Analysis

Fisher’s exact test was used to compare biochemical response to AA/P in subgroups. Progression-free survival estimates were calculated using the method described by Kaplan and Meier [45]. The log-rank test was used to compare time to biochemical progression in subgroups. Unadjusted and multivariable-adjusted logistic regression was used to assess the association between genetic variation and biochemical response. Unadjusted and multivariable-adjusted Cox proportional hazards regression was used to analyze the association between genetic variation and time to biochemical progression [46]. Multivariable-adjusted regression models included age at the time of AA/P initiation, Gleason Score at the time of initial diagnosis, prostate-specific antigen at the time of AA/P initiation, and whether AA/P was initiated pre- or post-chemotherapy in addition to the SNP of interest. Effect measures are expressed as odds or hazard ratio per minor allele. In order to assess associations at the SNP-level, the ProbABEL software was used to analyze the biochemical response and time to biochemical progression data using the imputed allele dosage [47]. In order to correct for multiple testing in the presence of linkage disequilibrium, a modified effective number of independent tests approach was employed [48,49,50]. The inferred *M*_eff_ for the four SNPs was 3 (adjusted α-level = 0.017). Data management and statistical analyses were performed using the SAS software (version 9.3, SAS Institute, Cary, NC, USA) and the R environment (R Foundation for Statistical Computing, Vienna, Austria). Graphs were created using the Stata software (version 13.1, StataCorp, College Station, TX, USA).

## 5. Conclusions

We identified the common genetic variant rs2486758 in *CYP17A1* that was negatively associated with a biochemical response to and time to biochemical progression in patients on AA/P with mCRPC. The identified variant is known to increase transcriptional activity, likely conferring resistance to AA/P through up-regulation of CYP17A1. These findings warrant external validation to verify the predictive power of this readily available biomarker.

## Figures and Tables

**Table 1:** Characteristics of 87 patients receiving abiraterone acetate and prednisone for metastatic castration-resistant prostate cancer.

*Demographics at the Time of Initial Prostate Cancer Diagnosis*	
Median age (years)	64 (43–89)
Median prostate-specific antigen (ng/dL)	7 (0–3229)
Stage	
T1–T2b	24 (28%)
T2c	25 (29%)
T3+	38 (43%)
N+	23 (38%)
M+	18 (56%)
Gleason grade	
5–6	11 (13%)
7	27 (31%)
8–10	49 (56%)
Radical prostatectomy	51 (71%)
***Patient Characteristics at the Time of Initiation of Abiraterone Acetate and Prednisone for Castration-Resistant Stage***	
Median age (years)	73 (54–90)
Median prostate-specific antigen (ng/dL)	66 (0–100)
Median body mass index (kg/m^2^)	30 (21–56)
Bone metastases	73 (84%)
Soft tissue metastases	43 (49%)
Previous androgen-deprivation therapy	87 (100%)
Previous docetaxel	56 (65%)
Previous enzalutamide	5 (6%)
Previous cabazitaxel	5 (6%)
Time from initial diagnosis (years)	8 (1–24)
Patients alive at the time of data analysis	38 (44%)

Data are given as median (range) or count (percent of the entire cohort).

**Table 2:** Association between common genetic variations in *CYP17A1* and biochemical response to abiraterone acetate and prednisone in 87 patients with metastatic castration-resistant prostate cancer.

SNP	*n*	Biochemical Response to Treatment		*p*
		Response Rates ^1^	Effect Estimate	
rs2486758	87	50%/17%	OR 0.22 (0.07–0.63)	0.005
rs4919685	87	38%/39%	OR 1.24 (0.64–2.40)	0.524
rs17115100	87	40%/37%	OR 0.82 (0.31–2.19)	0.689
rs743572	87	33%/40%	OR 1.13 (0.58–2.20)	0.718
		**Time to** **Biochemical** **Progression**		
		**Median Time to** **Progression ^1^**	**Effect Estimate**	
rs2486758	87	7.0 months/2.9 months	HR 2.23 (1.39–3.56)	<0.001
rs4919685	87	3.5 months/4.6 months	HR 0.82 (0.47–1.42)	0.429
rs17115100	87	4.5 months/5.3 months	HR 0.95 (0.67–1.35)	0.693
rs743572	87	3.5 months/4.6 months	HR 1.03 (0.73–1.46)	0.889

Effect estimates are expressed as unadjusted odds ratio (OR) (95% confidence interval) or hazard ratio (HR) (95% confidence interval) per minor allele. ^1^ Biochemical (prostate-specific antigen) response rates and median time to biochemical progression are listed as major homozygotes versus hetero- and minor homozygotes; SNP, single nucleotide polymorphism.

**Table 3:** Multivariable-adjusted effect estimates for rs2486758 and biochemical response to abiraterone acetate and prednisone as well as time to biochemical progression in 87 patients with metastatic castration-resistant prostate cancer.

Model	*n*	Response to Treatment	*p*
Unadjusted	87	OR 0.22 (0.07–0.63)	0.005
Age-adjusted (I)	87	OR 0.21 (0.07–0.62)	0.005
Multivariable-adjusted (II)	87	OR 0.22 (0.07–0.63)	0.005
Multivariable-adjusted (III)	87	OR 0.21 (0.07–0.62)	0.005
Multivariable-adjusted (IV)	87	OR 0.18 (0.06–0.57)	0.003
		**Time to Progression**	
Unadjusted	87	HR 2.23 (1.39–3.56)	<0.001
Age-adjusted (I)	87	HR 2.22 (1.40–3.55)	0.001
Multivariable-adjusted (II)	87	HR 2.26 (1.41–3.62)	0.001
Multivariable-adjusted (III)	87	HR 2.29 (1.43–3.67)	0.001
Multivariable-adjusted (IV)	87	HR 2.67 (1.64–4.36)	<0.001

Effect estimates are expressed as unadjusted and multivariable-adjusted odds ratio (OR) (95% confidence interval) or hazard ratio (95% confidence interval) per minor allele. Model I was additionally adjusted for age at the time of abiraterone acetate initiation. Model II was additionally adjusted for Gleason score at the time of initial diagnosis. Model III was additionally adjusted for prostate-specific antigen at the time of abiraterone acetate initiation. Model IV was additionally adjusted for prior administration of docetaxel.
